# Supplementary figures and images for: Ependymal polarity defects coupled with disorganized ciliary beating drive abnormal cerebrospinal fluid flow and spine curvature in zebrafish
Source: PLoS Biol. 2023 Mar 2;21(3):e3002008. doi: 10.1371/journal.pbio.3002008 (PMC10013924; doi:10.1371/journal.pbio.3002008)

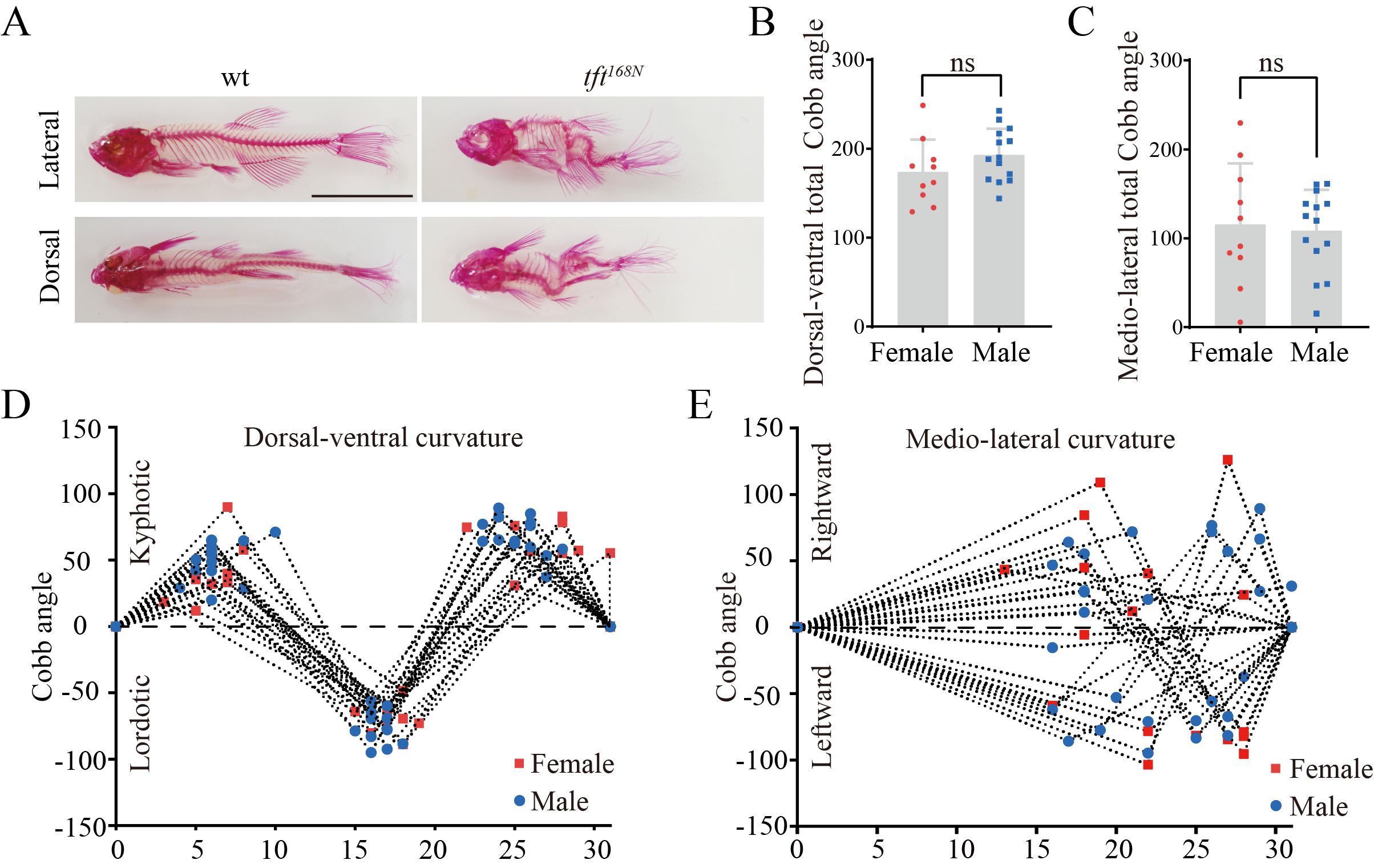

Supplement: S1 Fig — (A) Alizarin red staining results of wild type and ccdc57 mutant. (B-E) Statistical analysis and distribution pattern showing Cobb angles measured from dorsal-ventral and medio-lateral curvatures in female and male ccdc57 mutant zebrafish. Scale bar: 1 cm in panel A. The data underlying the graphs shown in the figure can be found in S1 Data. (TIF) [file pbio.3002008.s001.tif]

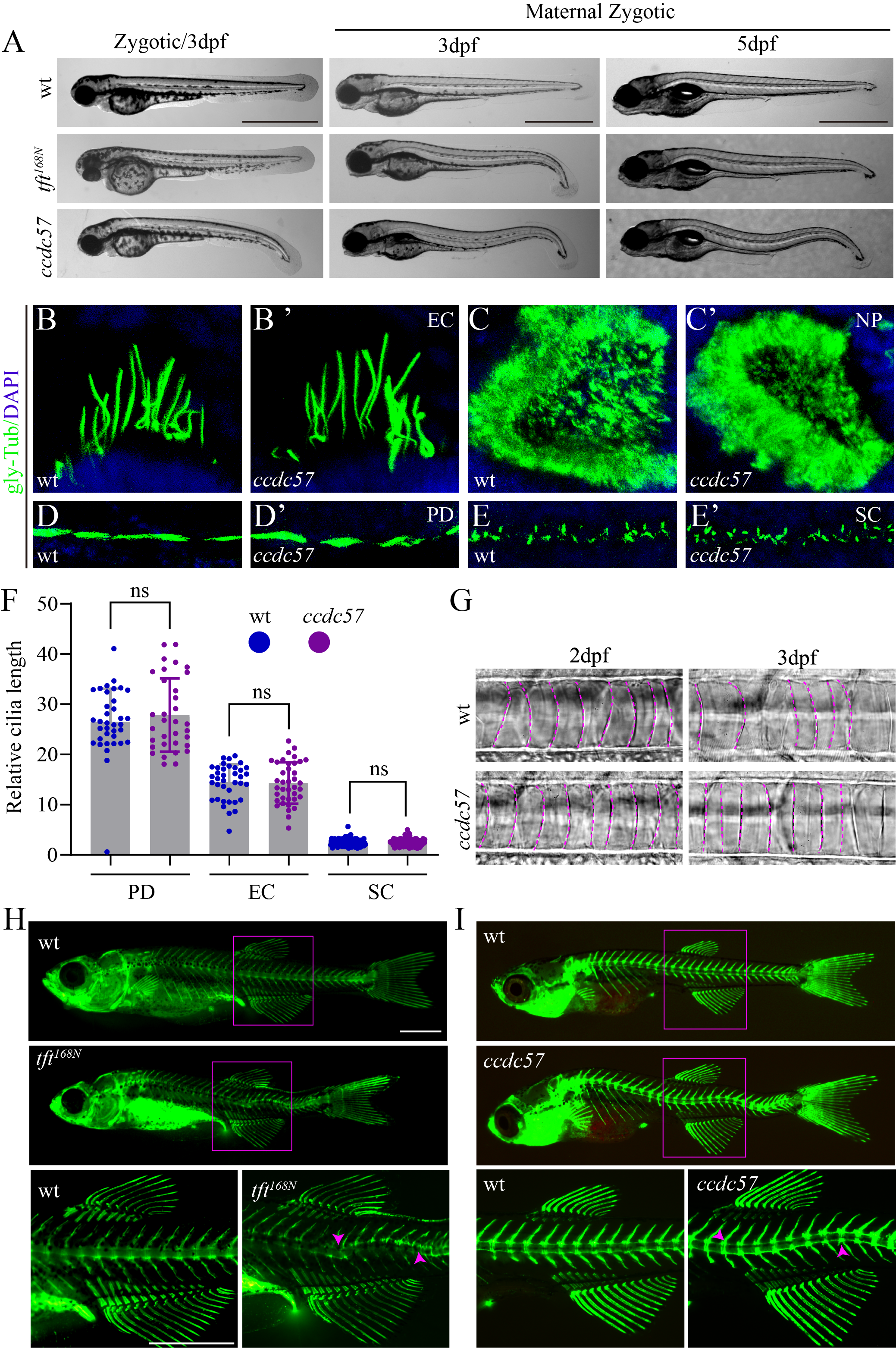

Supplement: S2 Fig — (A) External images showing zygotic and maternal zygotic (MZ) ccdc57 mutant zebrafish at indicated developmental stages. (B-E’) Confocal images showing cilia in the ear cristae (EC) (B-B’), nasal pit (NP) (C-C’), pronephric duct (PD) (D-D’), and spinal canal (SC) (E-E’) in 5 dpf wild type and ccdc57 mutants as indicated. Cilia were visualized with anti-glycylated tubulin in green, and nuclei were counterstained with DAPI in blue. (F) Statistical analysis showing relative cilia length in cristae, pronephric duct, and spinal canal. (G) Differential interference contrast (DIC) images of notochords in wild type and ccdc57 mutants. The purple dotted lines mark the margin of the neighboring notochord cells. (H) Fluorescent images showing calcein staining of 17 dpf wild type and ccdc57tft168N mutant zebrafish as indicated. (I) Fluorescent images showing the osteoblasts marked by Tg(Ola.Sp7:NLS-GFP) in 17 dpf wild type and ccdc57 mutant. Scale bars: 1 mm in panel A; 1 mm in panel H. The data underlying the graphs shown in the figure can be found in S1 Data. (TIF) [file pbio.3002008.s002.tif]

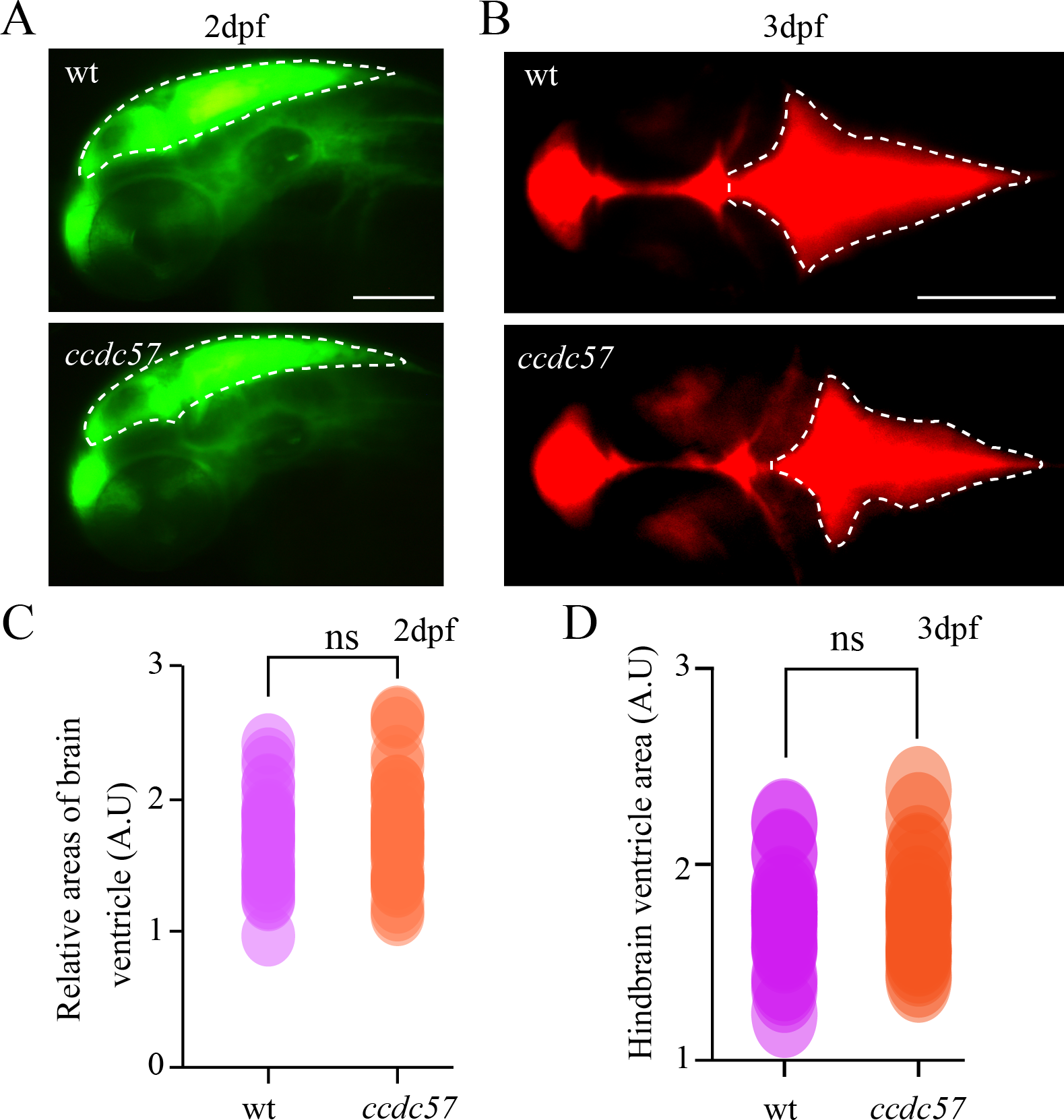

Supplement: S3 Fig — (A, B) Fluorescent images showing brain ventricles as indicated by injection of Rhodamine- or FITC-conjugated fluorescent beads (70 kDa) into zebrafish larvae at developmental stages as indicated. (C, D) Statistical analysis of brain ventricle size as indicated in panels (A) and (B). Scale bars: 50 μm in panels A and B. The data underlying the graphs shown in the figure can be found in S1 Data. (TIF) [file pbio.3002008.s003.tif]

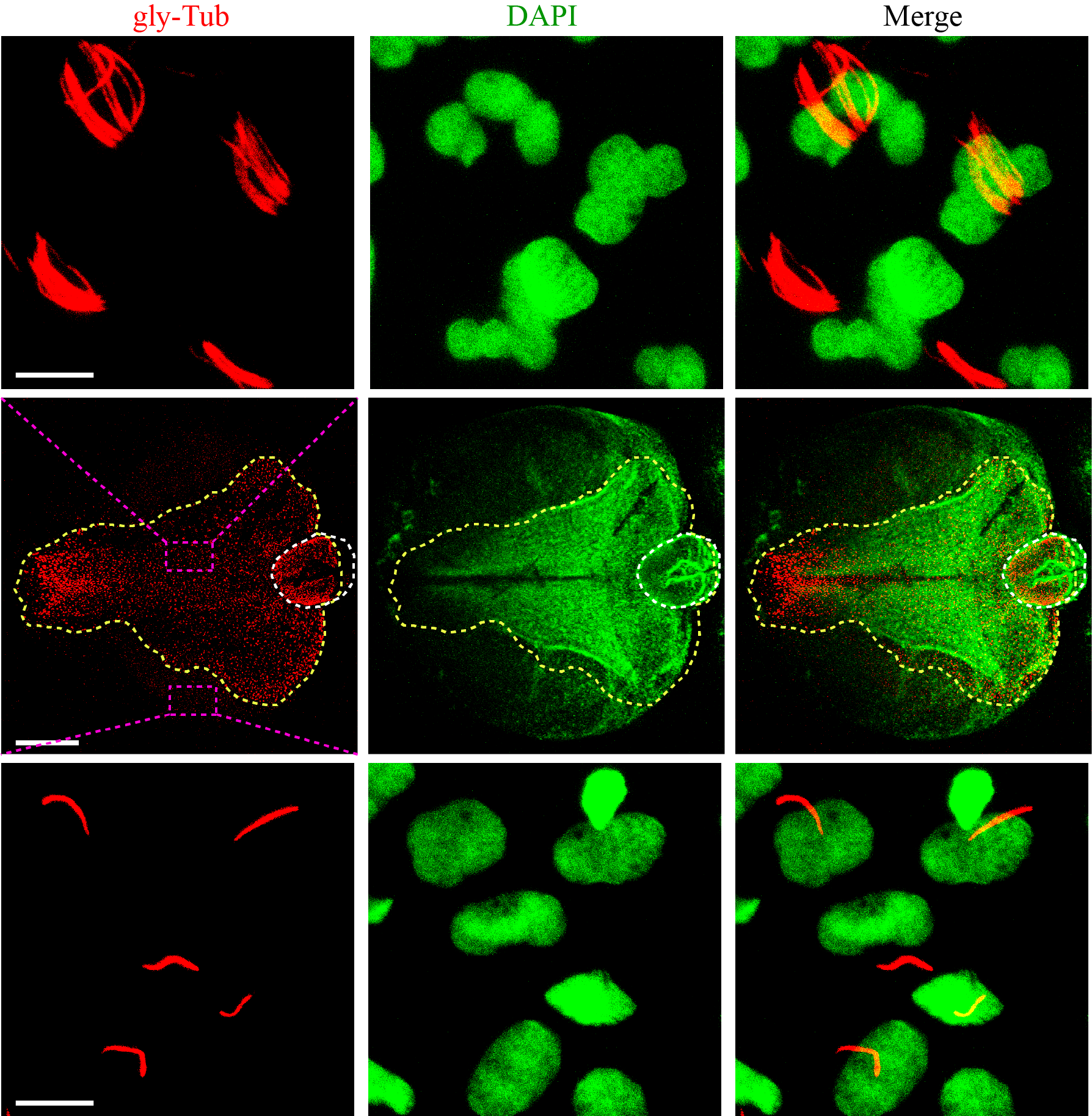

Supplement: S4 Fig — Confocal images showing the relative position of the ependymal layer and dChP (white dotted box) in the telencephalon. Multiciliated ependymal cells were enriched in the center region (yellow dotted box) and monociliated cells were located to the periphery. Cilia were visualized with anti-glycylated tubulin antibody (red), and nuclei (green) were counterstained with DAPI. Scale bars: 7.5 μm, 250 μm, and 7.5 μm from the top to the bottom. (TIF) [file pbio.3002008.s004.tif]

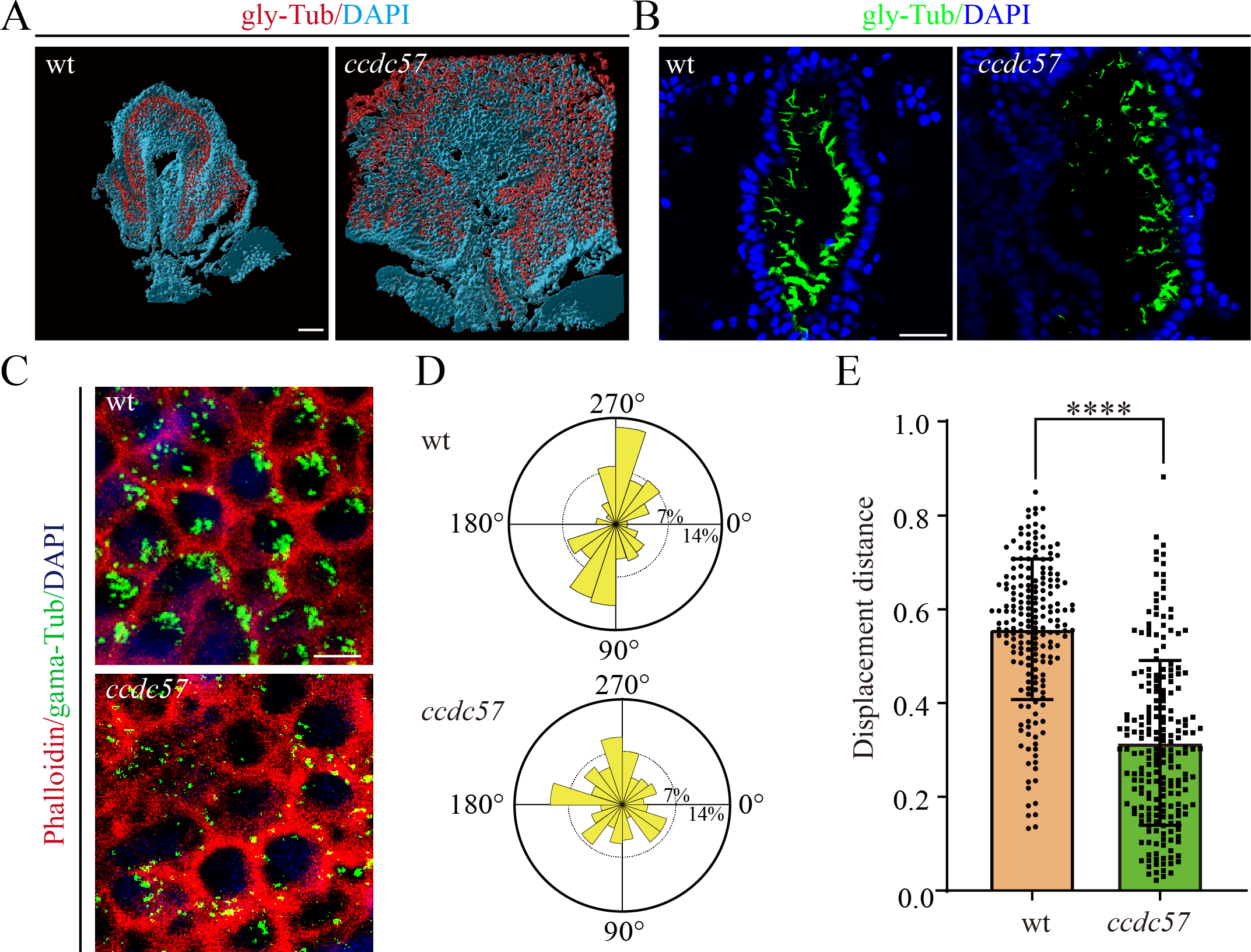

Supplement: S5 Fig — (A) Three-dimensional reconstruction of dChP showing the distribution of cilia (anti-glycylated tubulin, red) in wild type and ccdc57 mutant zebrafish. (B) Confocal images showing cilia are enriched in the center of the dChP folds as suggested from the 3D images. Cilia were visualized with anti-glycylated tubulin in green, and nuclei were counterstained with DAPI in blue. (C) Confocal images showing the distribution of the basal bodies in dChP of wild type and ccdc57 mutant. (D) Angular distribution of the basal bodies in wild type and ccdc57 mutants. (E) Statistical analysis showing the displacement distance of the basal bodies in wild type and ccdc57 mutants. Scale bars: 50 μm in panel A; 25 μm in panel B; 5 μm in panel C. The data underlying the graphs shown in the figure can be found in S1 Data. (TIF) [file pbio.3002008.s005.tif]

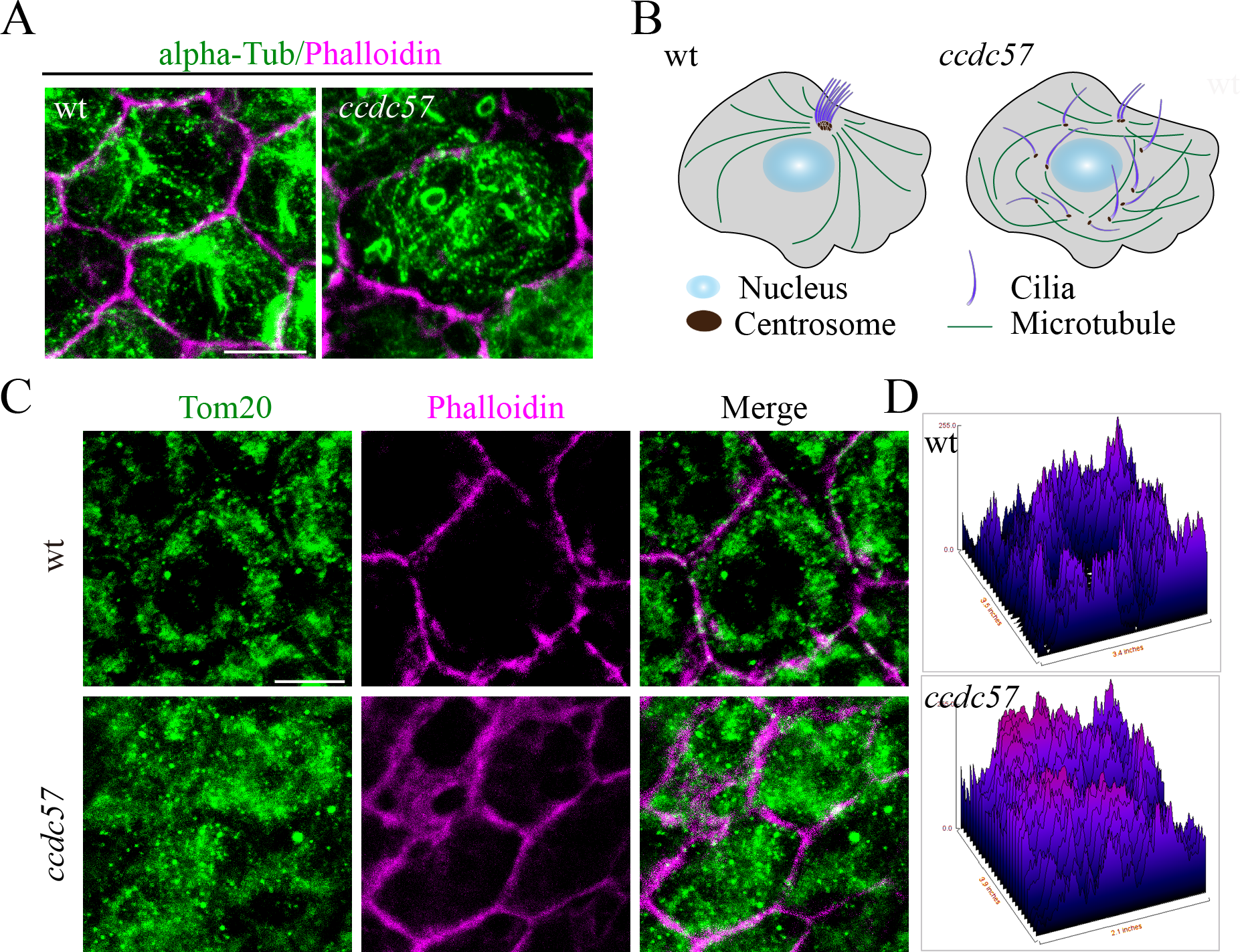

Supplement: S6 Fig — (A) Confocal images showing the microtubule network visualized with anti-alpha tubulin antibody in green. (B) Diagram showing the disorganized cilia and microtubule skeleton in the ependymal cells of ccdc57 mutant zebrafish. (C) Confocal images showing the distribution of mitochondria (Tom20) in wild type and ccdc57 mutant. In panels (A) and (C), Phalloidin was used to mark the peripheral cortex of the cell. (D) Plot images showing the distribution pattern of mitochondria in wild type and ccdc57 mutant. Scale bars: 7.5 μm in panel A; 5 μm in panel C. The data underlying the graphs shown in the figure can be found in S1 Data. (TIF) [file pbio.3002008.s006.tif]

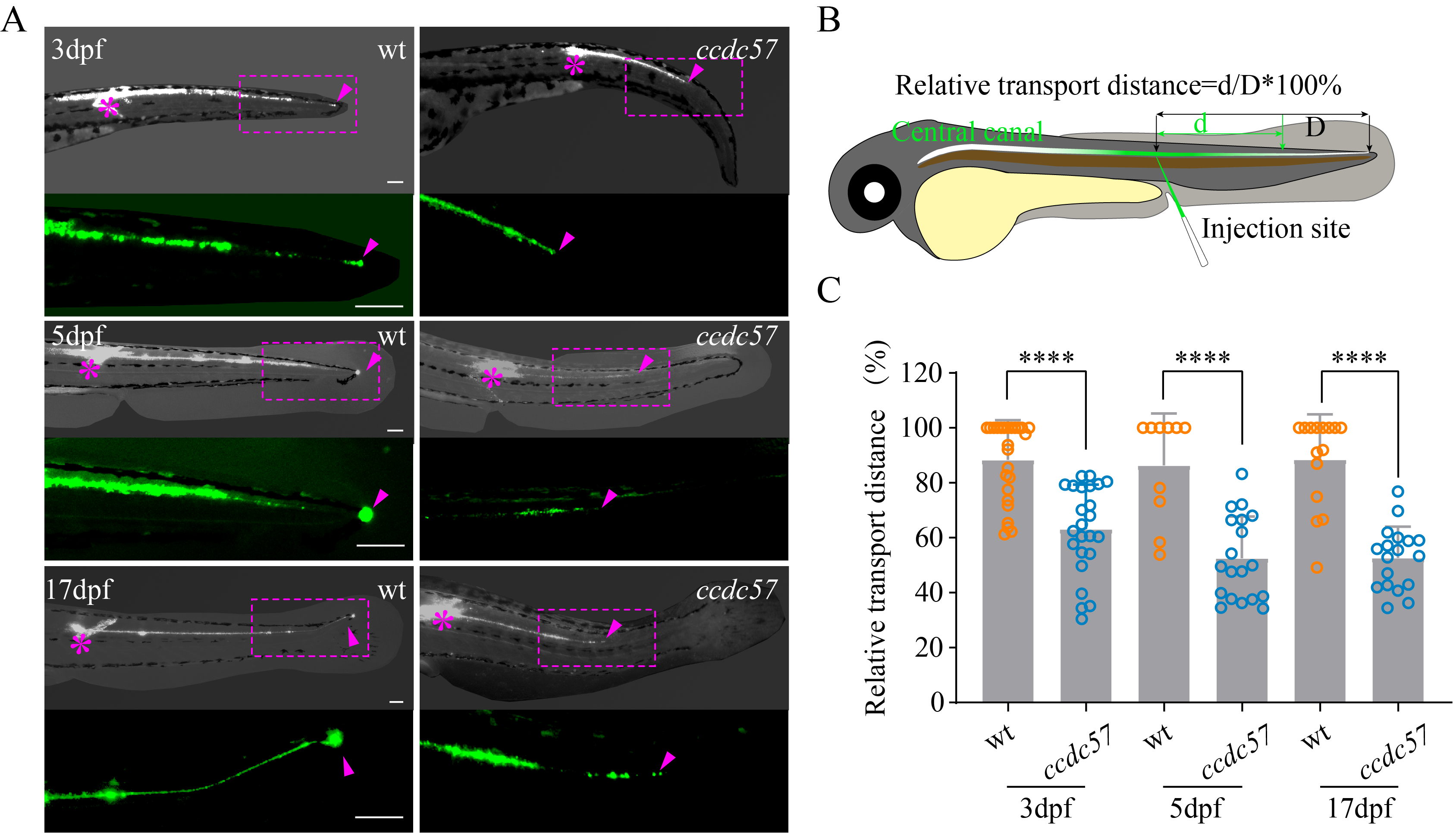

Supplement: S7 Fig — (A) Images showing the distribution of 100 nm fluorescent beads at 6 hour postinjection in wild type control or ccdc57 mutant larvae at different developmental stages. Enlarged views of the magenta boxed regions are shown below each panel. Asterisks indicate injection sites; arrowheads indicate the migration end of fluorescent beads in the central canal. (B) Model illustrating the calculation methods of relative transport distance of the fluorescent beads in the spinal canal. (C) Statistical analysis showing relative transport distances of injected fluorescent beads at 6 hour after injection. Scale bars: 10 μm in panel A. The data underlying the graphs shown in the figure can be found in S1 Data. (TIF) [file pbio.3002008.s007.tif]

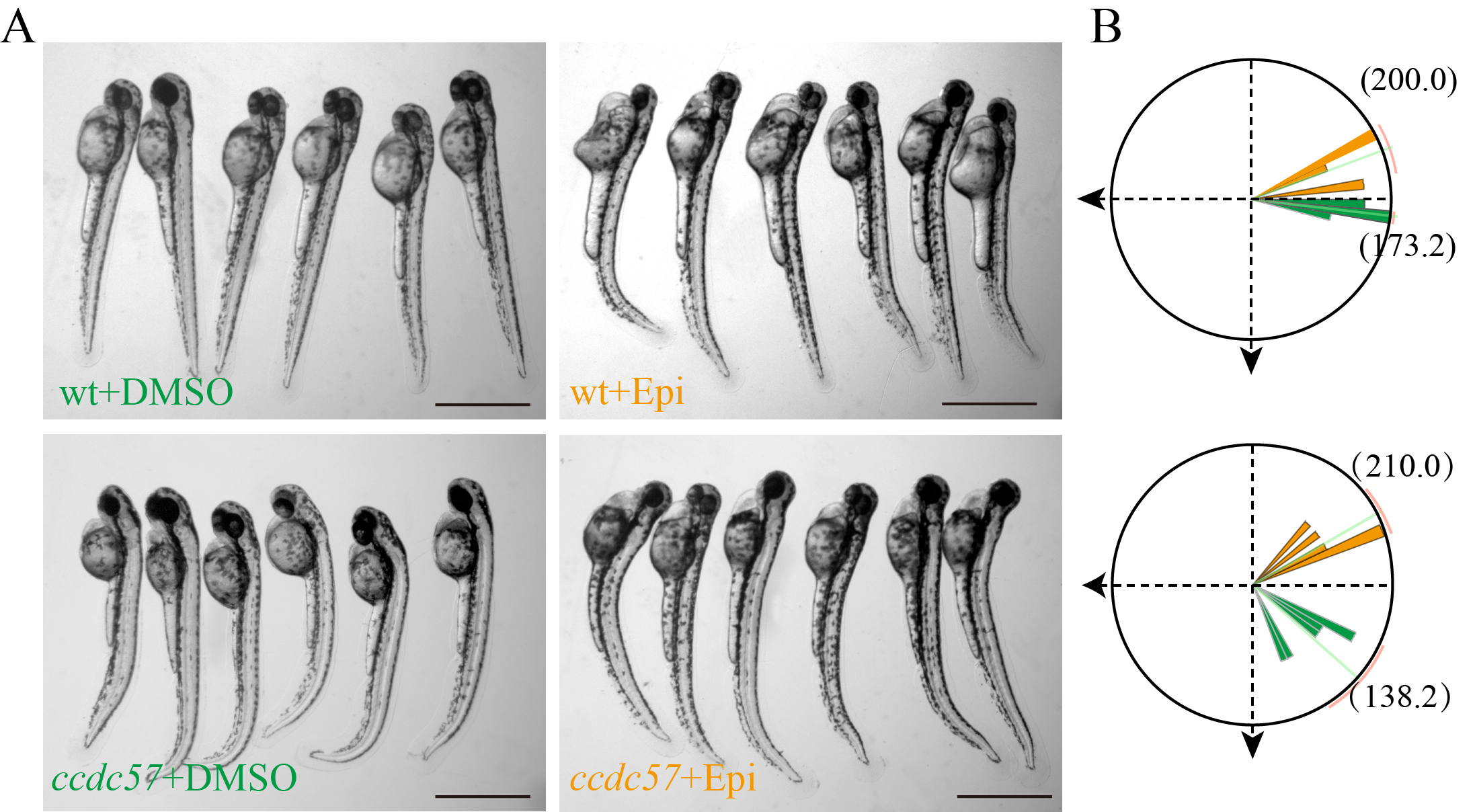

Supplement: S8 Fig — (A) Brightfield images of 3 dpf wild type and ccdc57 mutants treated with DMSO or epinephrine. (B) Statistical analysis of body curvature angles in wild type and ccdc57 mutants treated with DMSO or epinephrine. Scale bars: 1 mm in panel A. The data underlying the graphs shown in the figure can be found in S1 Data. (TIF) [file pbio.3002008.s008.tif]

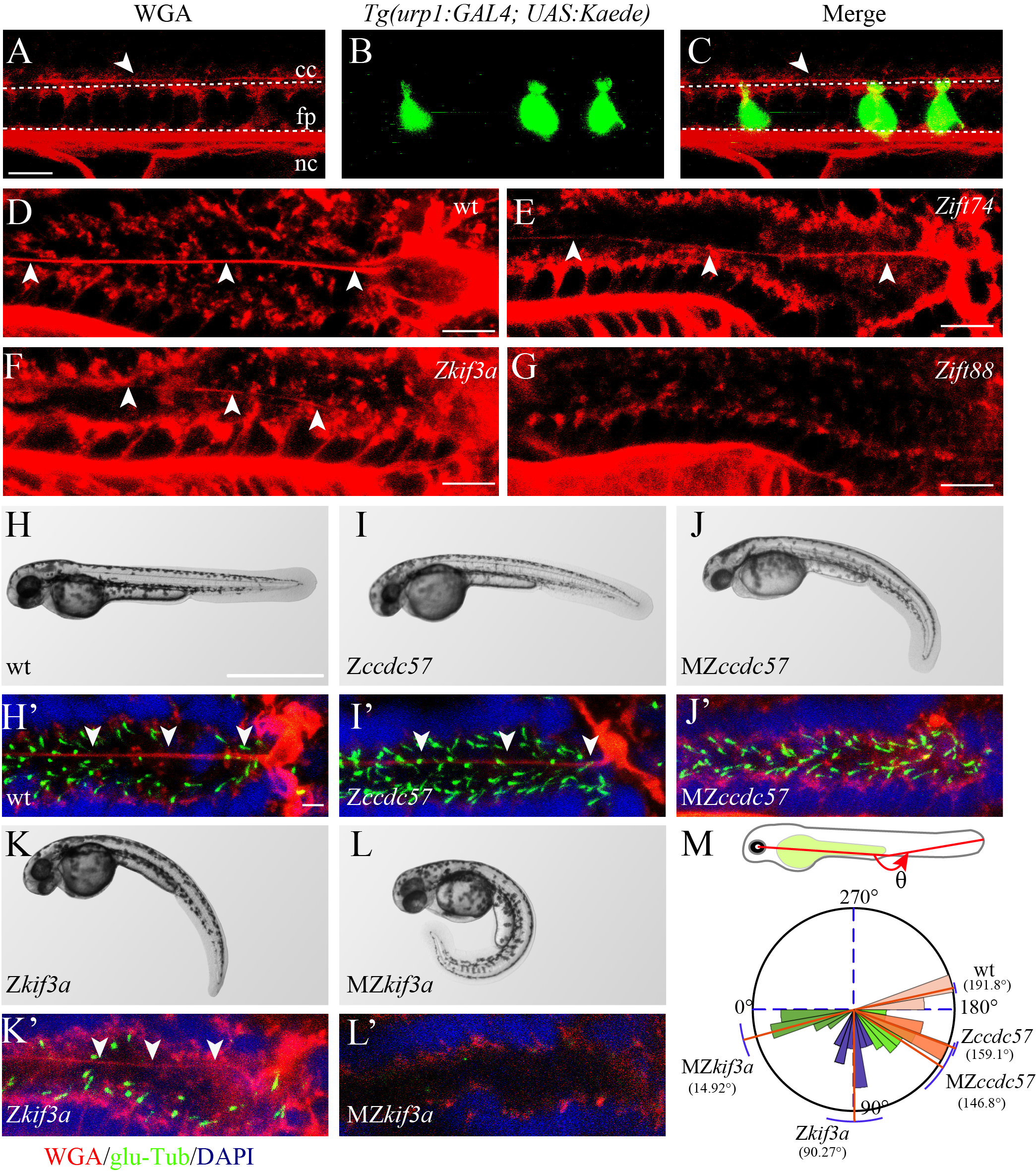

Supplement: S9 Fig — (A-C) Confocal images showing the relative position of RF (WGA, red, white arrowhead) and cerebrospinal fluid-contacting neurons (CSF-cNs, green) marked with Tg(urp1:GAL4; UAS:Kaede) in 48 hpf wild type zebrafish larva. (D-G) Confocal images showing the RF (arrows) in different cilia mutants at 48 hpf. (H-L’) External image of different cilia mutants as indicated at 48 hpf and confocal images showing the RF (white arrows) of indicates mutants. (M) Pie chart displaying the statistical analysis of average curved angle in different mutants as indicated at 48 hpf. The RF were labeled with WGA in red, cilia were stained with anti-polyglutamylated tubulin in green, and nuclei were counterstained with DAPI in blue. Scale bars: 10 μm in panels A-C; 5 μm in panels D-G; 1 mm in panels H-L; 5 μm in panels H’-L’. The data underlying the graphs shown in the figure can be found in S1 Data. (TIF) [file pbio.3002008.s009.tif]

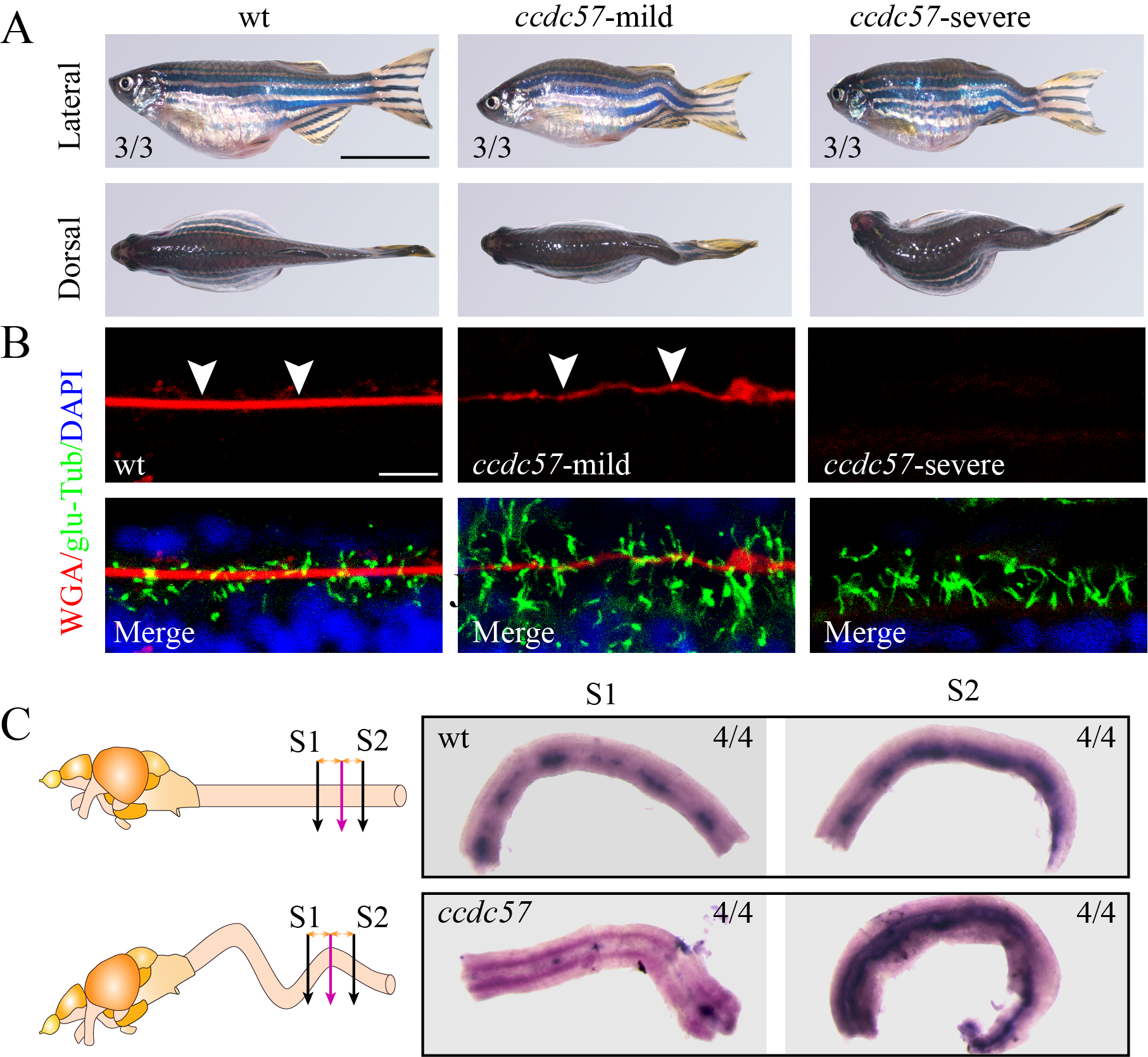

Supplement: S10 Fig — (A) The bright field images of wild type and ccdc57 mutants with different degree of scoliosis. (B) Confocal images showing the RF (white arrow indicated) in wild type and ccdc57 mutants. The RF was labeled with WGA in red, and cilia were stained with anti-polyglutamylated tubulin in green. Nuclei were counterstained with DAPI in blue. (C) Schematic representation showing the position of the spinal cord that was dissected for in situ hybridization analysis. The hybridization results were shown on the right. Scale bars: 1 cm in panel A; 10 μm in panel B. (TIF) [file pbio.3002008.s010.tif]

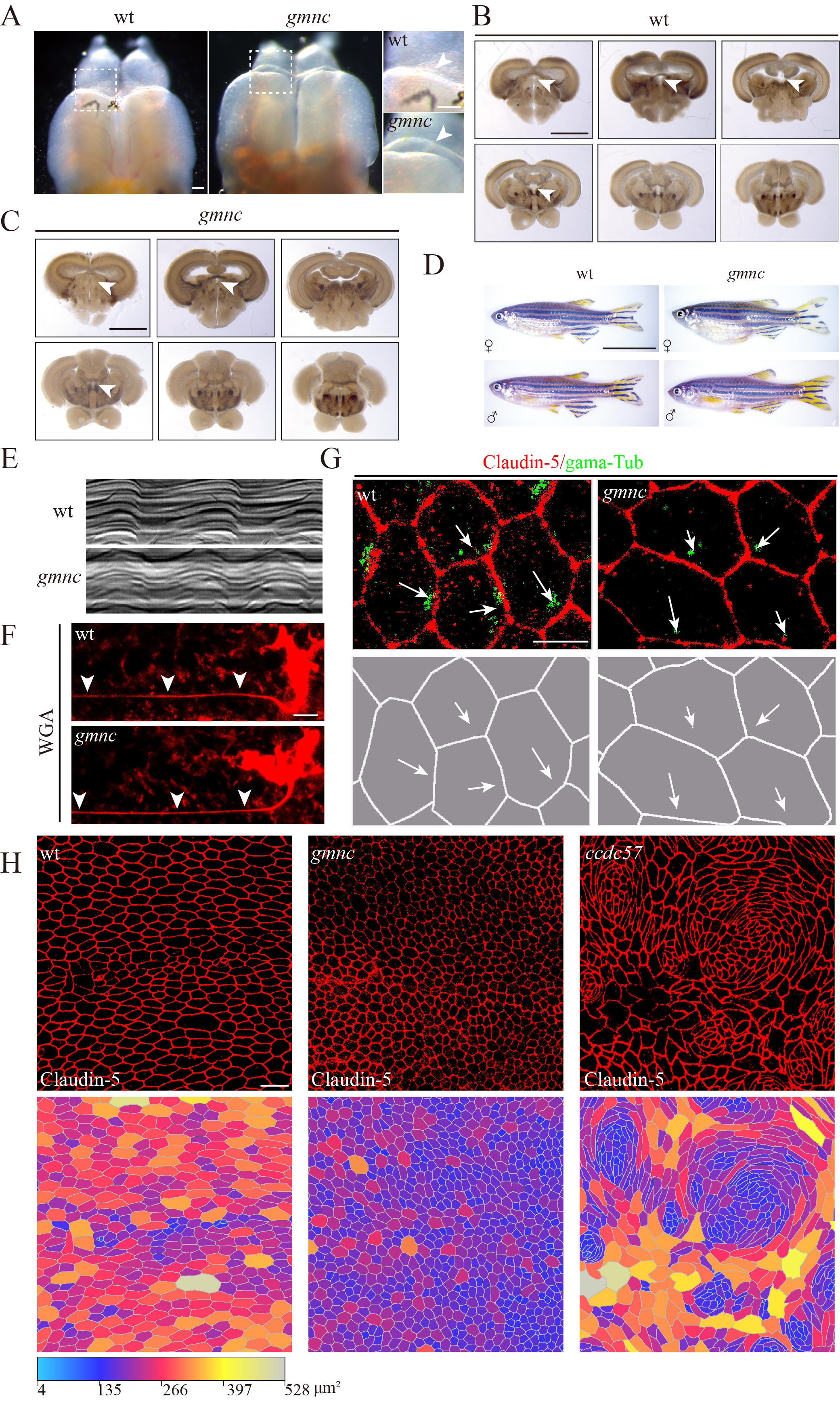

Supplement: S11 Fig — (A) External phenotypes of the telencephalon in wild type and gmnc mutant. (B, C) Cross sections of the brain ventricles in wild type and gmnc mutant. (D) Representative images of 3-months-old wild type and gmnc mutants. (E) Kymographs of cilia movement in the spinal canal of 5 dpf wild type and gmnc mutant larvae. (F) Confocal images showing the RF (red, white arrowhead) in wild type and gmnc mutant larva. (G) Confocal images showing the distribution pattern of basal bodies in the ependymal cells of wild type and gmnc mutant. The basal bodies were labeled with anti-γ tubulin antibody in green, and tight junctions were stained with Claudin-5 antibody in red. (H) Confocal images and schematic graphs showing the distributed pattern of ependymal cells in wild type, gmnc and ccdc57 mutants. Tight junctions were stained with Claudin-5 antibody in red. Scale bars: 100 μm in panel A; 1mm in panels B and C; 1 cm in panel D; 10 μm in panel F; 5 μm in panel G; 25 μm in panel H. (TIF) [file pbio.3002008.s011.tif]

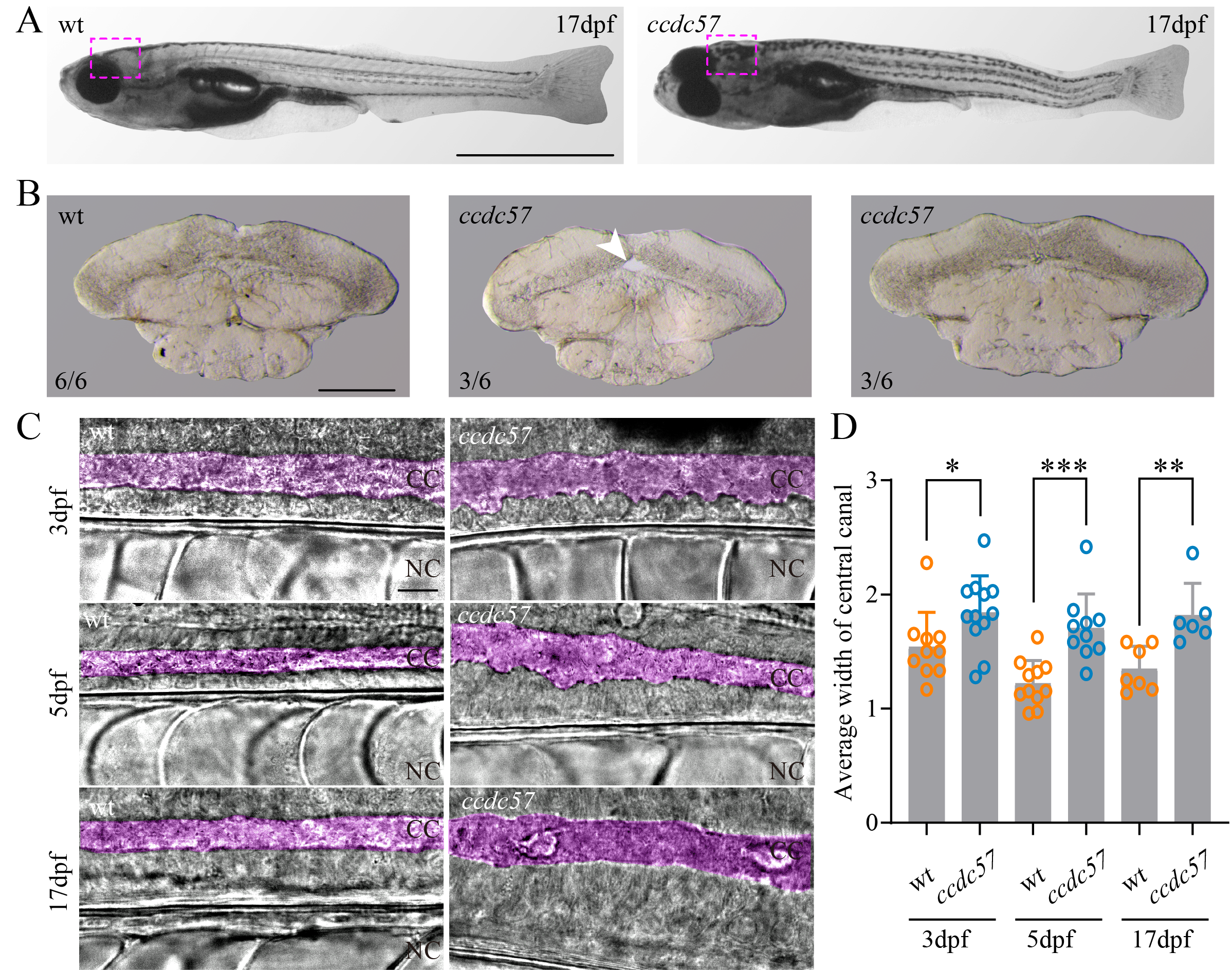

Supplement: S12 Fig — (A) External images of wild type and ccdc57 mutants at 17 dpf. (B) Cross-sections from the brain in wild type and ccdc57 mutants. The white arrowhead indicates hydrocephalus in some ccdc57 mutants. The number of dissected samples is shown in the bottom left. (C) Bright field images showing the central canal labeled with purple pseudo-color in wild type and ccdc57 mutants at different stages as indicated. (D) Bar graph with dots showing the width of central canal in wild type and ccdc57 mutants at different developmental stages as indicated. Scale bar: 2 mm in panel A; 200 μm in panel B; 10 μm in panel C. The data underlying the graphs shown in the figure can be found in S1 Data. (TIF) [file pbio.3002008.s012.tif]

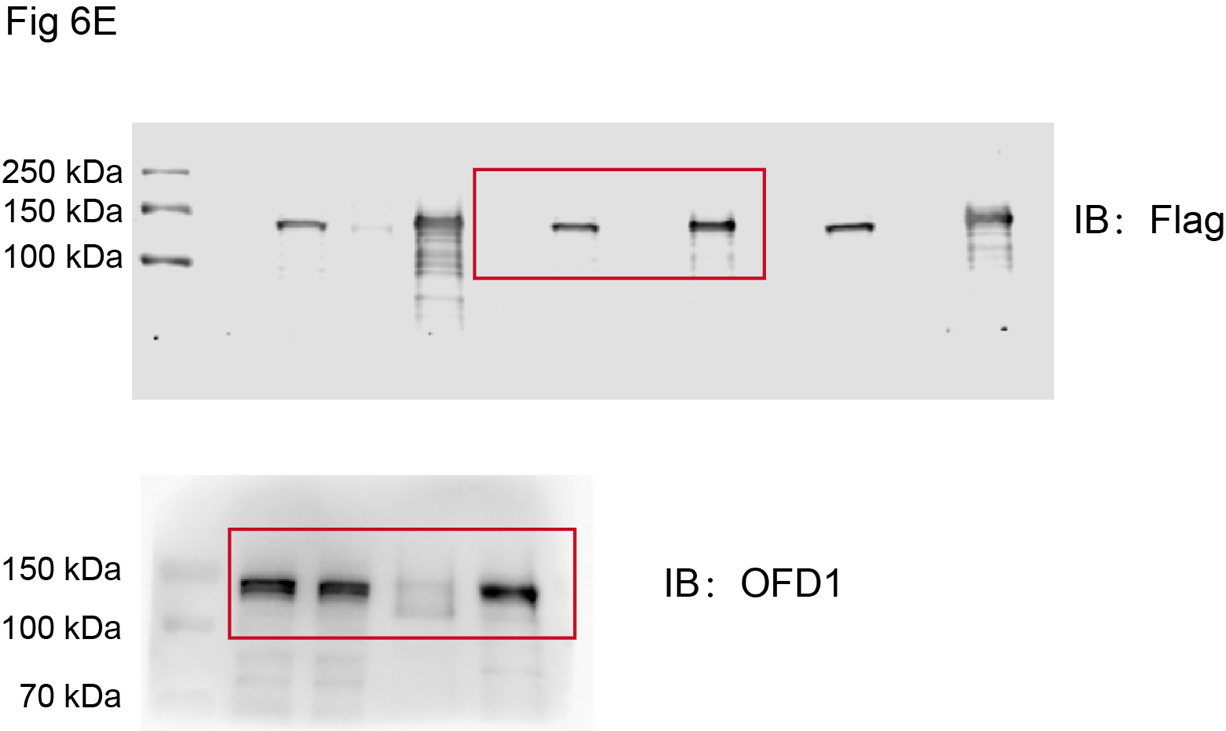

Supplement: S1 Raw Images — (PNG) [file pbio.3002008.s028.png]
