# Supplementary material for: Ependymal polarity defects coupled with disorganized ciliary beating drive abnormal cerebrospinal fluid flow and spine curvature in zebrafish
Source: PLoS Biol. 2023 Mar 2;21(3):e3002008. doi: 10.1371/journal.pbio.3002008 (PMC10013924; doi:10.1371/journal.pbio.3002008)
Supplement: Attachment — Submitted filename: Response to Reviewers.docx [file pbio.3002008.s029.docx]

**Reviewers' comments**

**Rev. 1:**

In line with previous results indicating that mutants with defective motile cilia exhibit deformations of the body axis and spine (Grimes et al Science 2016) because their Reissner fiber does not form (Cantaut-Belarif et al CB 2018) or is not maintained properly (Troutwine et al CB 2020; Rose et al CB 2020), the authors had shown previously that mutants leading to defective CSF flow showed scoliosis due to defects in the URP signaling expressed by CSF-contact neurons and acting on a receptor in the dorsal musculature (Zhang et al Nature Genetics 2018).

Here the authors provide new interesting evidence for yet another cilia related gene, the gene ccdc57 shown to be involved in the planar polarity of cilia, to be linked via different mutations to early onset curvature of the body axis (3dpf) and 3D torsion of the spine (17 dpf) as well as hydroencephalus. The authors link the mutations in this gene to changes in urotensin signalling, and show the first evidence for difference on the concave and convex side of urotensin signalling (via expression of the receptor UTS2R in the musculature) of AIS patients.

Major comments

A) The authors do not currently mention in their introduction & most of their results the large body of work performed by their colleagues to establish critical role of the Reissner fiber with CSF-contacting neurons as the axial sensory system critically-linking cilia function and URP peptides : the authors only mention it at the bottom of page 12 !! this is not fair to the literature and our understanding of the mechanisms that all converge on the fiber to confer a straight body axis. The authors should therefore mandatorily fix this in their revised manuscript by properly introducing the large body of work of the last 4 years.

The authors absolutely need to revise the introduction, the results and discussion whose first paragraph should point to the Reissner fiber as the link between the flow and the sensory neurons CSF-cNs that express the peptides.

- the Reissner fiber needs to aggregate the CSF flow that originates from motile cilia with caudal polarity in the central canal (Cantaut-Belarif CB 2018) ; consequently the lack of the Reissner fiber in 6 mutants with defective cilia explains deformation of the body axis (Cantaut-Belarif CB 2018)

- CSF flow is sensed by CSF-cNs via their coupling to the fiber (Orts Dell Immagine et al CB 2020)

- Based on the coupling of the fiber and CSF-cNs, URP peptides are expressed in CSF-cNs to act on downstream targets outside of the nervous system (Cantaut-Belarif eLife 2020)

- Monoamines (epinephrine, norepinephrine) that can rescue defects in the body axis bind to the Reissner fiber to act (Cantaut-Belarif eLife 2020).

- Similar roles of the Reissner fiber established for straightening the body axis in the embryo appear conserved in the juveniles, as defects in maintenance of the Reissner fiber at the juvenile stage lead to 3D deformation (Troutwine et al CB 2020; Rose et al CB 2020; Lu et al Biol Open 2020).

We thank the reviewer for these thoughtful suggestions and agree that all these findings are essential for our understanding of the role of Reissner Fiber during signal transmission and body axis development. In this revised manuscript we have expanded the introduction and main text to include all of these references and to further emphasize these important roles of the RF.

B) Here the authors found another gene involved in ciliary function whose mutations can lead, depending of the severity, to either deformation of the body axis at 3dpf or of the spine at 17 dpf. We therefore need them to investigate further :

1) - whether the different mutations lead to different CSF flow (not only cilia beating) at 3 dpf or 17 dpf ***using the refined tracking of fluorescent beads*** :

this should be quantified as done previously in the embryo using injections of fluorescent beads (Thouvenin et al eLife 2020; Thouvenin et al Bio-protocol 2021, <https://bio-protocol.org/exchange/protocoldetail?id=3932&type=0&searchid=1659333907627759&sort=0)>

the authors should elaborate whether there are fine difference in flow, especially for the mutant alleles leading to defects at 3 or 17 dpf respectively.

We thank the reviewer for these great suggestions. In the first version of this manuscript we showed subtle coordinated beating defects in the spinal canal cilia of the *ccdc57* mutants, while we did not observe significant changes in the brain ventricle sizes of the *ccdc57* mutant larvae at 2 and 3dpf. To further define the subtle defects in CSF flow, we performed fluorescent beads tracing experiments as suggested by the reviewer. By injecting 20 nm or 100 nm fluorescent beads into the central canal, we noticed bidirectional CSF flow, as showed previously, in the central spinal canal of wild type larvae at 30hpf and 3dpf (Movie S9, S11). Interestingly, the bidirectional CSF flow was still present in *ccdc57* mutants, while we also sometimes observed localized circular microparticle movements in the mutants (Movie S10, S12). Due to the increased thickness of the zebrafish trunk, tracking the movements of individual particles was not feasible at later stages (5 or 17 dpf). However, by injecting the 100 nm fluorescent beads into the middle of the central canal we were able to detect the transport of these fluorescent beads. In wild type 3, 5 and 17 dpf larvae, the fluorescent beads could easily be detected at the posterior end of the central canal 6 hour after injection (Fig S7). In contrast, the transport distances were significantly reduced in the *ccdc57* mutant spinal canal at 3, 5 and 17 dpf, and none of the *ccdc57* mutant larvae exhibited fluorescent beads at the end of the central canal (Fig S7).

Here, we also have compared the width of the central canal between wild type and *ccdc57* mutants. We found that the central canal was significantly wider in the *ccdc57* mutants at 3, 5 and 17 dpf (Fig S12C-D). Together, these data suggest that loss of Ccdc57 resulted in mild CSF flow defects and enlarged central canal, due to uncoordinated beating of spinal canal cilia.

2) - whether the severity of the morphogenetic defects are correlated with different level of defects in the formation or maintenance of the Reissner fiber - this is critical and not pursued enough。

To clarify the relationship between the Reissner fiber and body axis defects, we first investigated Reissner fiber in *ccdc57* mutants exhibiting different scoliosis severity. We found clear evidence that defects in Reissner fiber assembly were closely related to the severity of scoliosis (Fig S10A-B). Moreover, we showed that RF defects were also related to the severity of body curvature in both *ccdc57* and *kif3a* mutants at larvae stages (Fig S9H-M).

3) - how to explain why the different mutations of ccdc57 lead to distinct effects on the CSF flow & on the Reissner fiber formation/maintenance.

As we mentioned in the first manuscript version, the *tft^168N^* allele introduced a stop codon in the 11th exon of *ccdc57*, resulting in the truncation of the final 407 C-terminal amino acids, while the N-terminal 572 amino acids were still encoded by the *tft^168N^* mutant allele, and may be partially functional. In contrast, the *ccdc57^Δ7^* mutants harbored a mutation in the first exon, which eliminates most of the amino acids of Ccdc57 protein. To further investigate this, we performed qPCR analyses on the expression of various *ccdc57* mutant alleles. We found that the expression of the *tft^168N^* allele was largely maintained in the mutants, while expression of *ccdc57^Δ7^* was significantly decreased due to nonsense mediated decay. These results suggested that, compared to the *ccdc57^Δ7^* mutants, the *tft^168N^* may be a hypomorphic mutation due to the presence of the first 572 amino acids, which may account for the varied severity observed in different *ccdc57* allele mutants.

As for the variable severity of scoliosis and RF assembly defects observed within the same mutant line, we think that several factors may be involved. First, maternal proteins play a vital role during early development, as demonstrated by the fact that *MZccdc57* mutants display stronger body curvature than observed in zygotic *ccdc57* mutants. In addition, the amount and half-life of maternal proteins may differ between sibling larvae. In several cilia mutant lines, we could observe the maternal protein effects in zebrafish aged as late as one month. It is possible that the maternal effects of Ccdc57 are different among individual larvae, resulting in variable defects in RF assembly. Secondly, albeit with lower transport efficiency, CSF flow was largely maintained in *ccdc57* mutants. Multiple factors contribute to CSF flow, such as heart beating, body movements and swimming behavior (Olstad, Ringers et al., 2019). It is likely that these factors may variably affect CSF flow, an in turn variably regulate the assembly of RF. Finally, we think that genetic background, feeding behavior and growth environment may also contribute to the variable severity observed in *ccdc57* mutants.

4) - whether the effects on hydroencephalus was always correlated and possibly precedes or follows defects in the Reissner fiber formation/maintenance.

We believe that both hydrocephalus and RF defects are caused by abnormal CSF transport due to *ccdc57* mutant ciliary motility defects. We could observe RF defects in *ccdc57* mutants as earlier as 3dpf, while we did not observe obvious hydrocephalus at this stage. However, we did notice the enlarged spinal canal at this stage (Fig S12C-D). In our opinion, RF assembly is more sensitive to abnormal CSF flow, while hydrocephalus develops due to the longer term accumulation of CSF flow defects in *ccdc57* mutants. Indeed, we could only detect hydrocephalus in some *ccdc57* mutants at 17 dpf (Fig S12A-B).

C) The authors establish a correlation between expression of urp2 and curvature of the spine.

1) They should quantify the segment number corresponding to the site of maximal deformation as well as the graded expression of the peptide.

Thanks for these great suggestions. To further clarify the relationship between spinal curvature and urotensin neuropeptide expression, we carefully dissected the spinal cord, cut it into several fragments, and only selected those fragments with severe curvature for *in situ* hybridization analysis. Moreover, we also cut the curved spinal cord into two segments at the apex (Fig S10C), and showed that the expression of *urp2* was enriched in the posterior portion of the curved fragments in *ccdc57* mutants, while much less expression was detected in other locations of the spine. These results suggested that the spinal curvature in *ccdc57* mutants is closely related to the local upregulation of *urp2* neuropeptides.

2) In the same line, we would expect them to infer a novel mechanism at play to link local urp expression and curvature: this new evidence contrasts with the postulated action of URP acting broadly on the urts2r3 receptor in the dorsal musculature. How can the peptide have a restricted action if its target are widespread on the dorsal musculature ?

We agree with this reviewer that the local control of spinal curvature by Urp2 neuropeptides is somewhat different from previous hypotheses. Because the Urotensin receptor is mainly expressed in the muscle fiber cells, it is unlikely that urotensin neuropeptides will be delivered directly to the muscle cells through synaptic transmission, considering that there are relatively few CSF-cNs. In contrast, we think that after release, Urotensin neuropeptides may diffuse for a long distance within the extracellular space before reaching their receptors in the muscle fibers. As zebrafish larvae grow, the diffusion distances become relatively shorter as the spine gradually elongates. Thus, we hypothesize that individual CSF-cNs may release the neuropeptides and control muscle fiber contraction only in a relatively small region, and the uneven distribution of Urotensin may cause locally overactivated muscle contraction resulting in spinal curvature.

Minor comments

- stage of the fish should be given in mm as well as dpf in different facility / with different feeding protocol will not lead to the same stage

Yes, we agree with this comment and have added scale bars to indicate the size of the zebrafish.

**Rev. 2:**

The focus of this manuscript by Xie et al is scoliosis developed in zebrafish ccdc57 mutants. In addition to morphological phenotypes, the authors provide evidence that the mutants show rotational, translation and tissue PCP defects in multi-ciliated ependymal cells.

Ccdc57 has been linked to centrosomes and cilia before. However, this is the first time ccdc57 is inked to scoliosis. Multiple additional interesting and novel results were presented, including hydrocephaly, disorganized cilia and planar polarity phenotypes at multiple levels in ccdc57 mutants. However, the manuscript is quite diffuse. The authors made multiple conclusions, yet the rationale of each experiment and the connection between different experiments is not always clear. For example, in zebrafish ciliary mutants, it is thought that changed urotensin expression leads to abnormal signaling through urotensin receptors specifically expressed in dorsal slow-twitch muscles. Here the authors show that in idiopathic scoliosis patients, the expression of UTS2R is higher in the convex side. Do the authors suggest that abnormal and somehow randomly sided expression of UTS2R is causal for scoliosis in these patients? This may be an interesting mechanism, yet how it relates to the rest of the manuscript is not clear.

Another example is figure S9 and the discussion on gmnc. A previous study using a temperature sensitive mutant suggests that there is a time sensitive window for developing scoliosis in juvenile zebrafish. This time window coincides with a growth spur and the stage ccdc57 mutants develop significant axis phenotypes. In contrast, gmnc is mostly involved in the biogenesis of multi-cilia, which occurs in ependymal cells after this time window. In this context, the comparison between gmnc and ccdc57 mutants is not particularly revealing. As a result, a clear and unifying mechanism is lacking. A more focused approach and investigating a potential role of ccdc57 in the basal foot will strengthen this manuscript. Below is a list of specific points.

We thank the reviewer for these thoughtful comments. Our zebrafish studies suggest that abnormal regulation of urotensin signals contributes to scoliosis development. We therefore wanted to further test whether the Urotensin signaling pathway is altered in AIS patients. Surprisingly, although we did not find changes in expression of Urotensin neuropeptides in human AIS patients, we found upregulated *UTS2R* expression in the convex curves of the spine. We agree with this reviewer that this may indicate novel mechanisms of scoliosis development that need further investigation beyond the scope of this study. In our opinion, it is possible that human scoliosis develops due to an imbalance in the contraction of muscle fibers surrounding the spine, as occurs in zebrafish. Although it is difficult to determine at this time whether altered UTS2R expression is a causative factor for scoliosis, we think that abnormal Urotensin signaling may contribute to the progression of scoliosis.

As for the *gmnc* mutants, we agree with the reviewer that multicilia develop later in the ependymal cells, and is therefore is not likely to be causative for scoliosis. Here we use the *gmnc* mutant data to demonstrate that scoliosis is not due to the absence of multicilia, but rather to uncoordinated cilia beating caused by ependymal polarity defects. Scoliosis was not observed in *gmnc* mutants due to the fact that ependymal polarity was largely maintained in these mutants. However, in *ccdc57* mutants, ependymal polarity defects were observed as earlier as 17 dpf, which we believe is the major cause for the development of scoliosis in *ccdc57* mutants.

Again, we thank the reviewer again for these helpful suggestions which we have incorporated in this revised manuscript to make these points more clearly.

1. The title is very broad and does not convey the specifics of the manuscript well.

We have changed the title of this manuscript to make it more specific.

2. It was shown that flow is not defective in ccdc57 mutants at the embryonic stage. Is this still true at the stage when mutants start to develop scoliosis? This is significant because directional flow could entrain PCP.

We thank the reviewer for this excellent question. In the original version of this manuscript, we showed that CSF flow is grossly normal in *ccdc57* mutants, although we cannot exclude the presence of subtle defects in CSF flow. To further explore whether CSF flow is defective in *ccdc57* mutants, we performed fluorescent particle tracing experiments. By injecting 20 nm or 100nm fluorescent beads into the central canal, we observed bidirectional CSF flow in both control wild type and *ccdc57* mutant zebrafish (Movie S9-12). Although there was no significant change in the direction of particle movements, we noticed that transport distances of the fluorescent beads were significantly reduced in *ccdc57* mutants (Fig S7). While the injected fluorescent beads were transported to the end of the caudal spinal canal in most wild type larvae, none of the mutants contained any particles in these region (Fig S7). Moreover, we found that the width of the spinal canal was significantly enlarged in *ccdc57* mutants at early developmental stages (Fig S12C-D). Together, these data suggest that *ccdc57* mutants exhibit relative minor CSF flow defects, which result in PCP defects at later stages of development.

3. Introduction: "We provide data showing that Ccdc57 is crucial for the establishment of planar polarity of ependymal cells, whose defects are probably the major reason of scoliosis formation in ciliary mutants". This statement seems overstretched.

Thank you for this comment - we have made edits to avoid this overstatement.

4. Text regarding Fig 1D, "two alleles complemented each other" should be "two alleles failed to complement each other"

Thank you for this careful review – we have corrected this statement.

5. Text regarding different alleles of ccdc57 "Moreover, maternal Ccdc57 protein may contribute to early embryonic development in the tft168N mutants." Maternal protein will contribute to early development regardless genotype.

Thank you for this careful review – we have corrected this statement.

6. Figure 2. Please provide sample orientation for H and I, and explain H' and I'

These panels are still images from the Movie S1. The 3D reconstruction movies were generated from serially focused images from the diencephalic choroid plexus. These two images were shown as ventral side views, viewing from the brain tissue to the dorsal epithelia. We have added this description to the Figure Legend and also in the Materials and Methods Section for 3D reconstruction. The H’ and I’ panels were simulated 3D reconstructions. Since they are very similar to panels H and I, we have removed these two images in this revised Figure 2.

7. Figure 3. Please provide the age of fish analyzed

Thank you for this comment. We have edited this revised manuscript to state that the Figure 3 results are from 3 month old adult zebrafish.

8. Basal body localization and cilia orientation are not affected in ccdc57 mutants at larvae stages. Please discuss

Although the apical distribution of basal bodies was not altered in *ccdc57* mutant larvae, the coordinated beating of cilia was indeed defective. We showed that the tilting direction and rotation angle of cilia were dramatically changed in *ccdc57* mutant larvae (Fig 5E-G), while the length and beating frequency remained normal (Fig S2E-F, 5D). Because CCDC57 is a satellite protein localized to the pericentriolar material, it is likely that this protein may regulate the orientation of basal bodies, including the direction of the basal foot, thus controlling the orientation of cilia beating. However, it is difficult to investigate this in zebrafish larvae. As far as we know, one of our colleagues conducting fine structure analyses of PCM proteins found that knockdown of CCDC57 in mouse ependymal cells altered the direction of basal foot (Personal Communication). We have added this in the Discussion section in this revised manuscript.

9. Movies need better annotations with info regarding sample orientation, size scale and playback speed. Movie S7 shows almost pulsative movement of cilia, which is not obvious in S9. However, it is difficult to draw conclusions, especially for coordination between cilia, by visual inspection. Kymographs will be helpful.

We thank the reviewer for this suggestion. We have added the orientation, scale bar and playback speed in these movies. We also have updated the original Movie S9 (currently Movie S13). We have also added kymographs of these movies to Fig 5C and Fig S11E.

10. Discussion: "Ccdc57 may orchestrate the polarity of the microtubule network downstream of PCP signals, and Ccdc57 mutations may cause abnormal organization of the microtubules and thereby disrupt proper cell polarity and the distribution of basal bodies." Related to this, Figure S6 shows gross disorganization of microtubule, not necessarily the more subtle PCP of microtubules. It is also possible that defective basal foot disrupts the localization of basal bodies and subsequently the microtubule network.

Yes, we agree with these excellent points. We think it is likely that Ccdc57 regulates the direction of the basal foot, thus changing the polarity of the microtubule network and the orientation of ciliary beating. Ccdc57 has recently been shown to exhibit microtubule binding activity. It is therefore possible that Ccdc57 may regulate microtubule network directly. We have added this discussion in the revised manuscript.

**Rev. 3:**

This is a highly comprehensive look at the ccdc57 mutant zebrafish which exhibits phenotypically hydrocephalus and scoliosis. The downstream experimentation secondary to mutations within this animal model are well described including issues with the cilia, polarity and urotensin signaling. The article is well written and has significant figures within the main manuscript and supplemental material that support the overall interpretation that mutations within this gene cause ciliary defects resulting in differences in ciliary action, cellular polarity and potentially CSF flow. Phenotypically hydrocephalus and scoliosis develop in addition to a disorganization of the RF which has been shown to be of importance in spinal development.

I raise the following questions for the authors that could be addressed or clarified:

what is the role of ccdc57 within the mitochondria?

We appreciate these excellent comments. We believe that Ccdc57 may not have a direct role on mitochondria function, although defects in Ccdc57 alter the overall distribution of mitochondria. It is well known that mitochondria are distributed by microtubule-based transport systems. We used the abnormal distribution pattern of mitochondria (via Tom20 staining) to further show the polarity defects of the microtubule network in ependymal cells of *ccdc57* mutants.

Relation between the disorganization of the RF and the Ccdc57 gene mutation causing ciliary issues. What element comes first in the formation of the spinal curvature?

We think both RF assembly and CSF flow defects are caused by uncoordinated beating of cilia in the spinal canal of *ccdc57* mutants. It was previously shown that RF assembly requires motile cilia to drive CSF flow (Cantaut-Belarif, Sternberg et al., 2018). It is likely that ciliary defects in *ccdc57* mutants cause abnormal CSF flow, thus affecting the assembly of RF, which in turn affects the activation of downstream Urotensin signals. To further prove this, we compared RF assembly in *ccdc57* mutants with both severe and milder scoliosis, showing that RF defects were more severe in mutants with strong scoliosis. We have included these data in Fig S10 in the revised manuscript.

There is significant detail as to when the scoliosis is observed within the mutants versus the wild type, but I could not find the timing in which the hydrocephalus appeared (see figure 2). does this happen simultaneously or at differing timepoints?

Thank you for raising this interesting question, also raised by reviewer 1. In *ccdc57* mutants, hydrocephalus developed due to CSF flow defects caused by uncoordinated cilia beating. However, we think CSF flow defects are relatively minor as compared to ciliary mutants with paralyzed cilia (Zhang, Jia et al., 2018). To further characterize this, we monitored the movement of 100nm fluorescent beads injected into the central canal. Compared to WT control larvae, we found that average transport distances were significantly shorter in *ccdc57* mutants (Fig S7). Moreover, the width of spinal canal was enlarged in *ccdc57* mutants (Fig S12C-D). We also showed that there was no obvious hydrocephalus in 2 or 3dpf larvae. But when we examined 17 dpf *ccdc57* mutant zebrafish, we found that while only a small number of mutants developed hydrocephalus at this time, they all eventually developed scoliosis (Fig S12A-B). Together, we think hydrocephalus formed due to the accumulative effects of abnormal CSF flow, and develops gradually as the *ccdc57* mutant larvae grow.

There are other models of scoliosis within the zebrafish - many with the associated hydrocephalus and RF disorganization. There is one model related to Kif7 that reportedly does not have either of these issues. This is also a ciliary protein with a dual function within the cilia and the hedgehog signaling pathway. Can the authors hypothesize this type of model that may exhibit scoliosis 'downstream' of other phenotypic observations?

We are familiar with the phenotypic description of *kif7* mutants by Terhune et al. We think it is likely that scoliosis development in this mutant line is also due to ciliary disfunction. As the author mentioned, 6–25% of *kif7* mutant larvae displayed body curvature, a classic phenotype of cilia mutants. The length of cilia was also significantly increased in the mutants (Fig 5F) (Terhune, Cuevas et al., 2021). As the author did not provide analysis of cilia motility, it is difficult to determine whether cilia motility is normal in these mutants. Moreover, it remains unknown whether hydrocephalus and RF assembly defects develop at later stages. In our hands, we showed that zebrafish with minor ciliary defects, similar to those in *kif7*, displayed normal RF assembly at 5 dpf. However, RF is highly disorganized in the adult mutants exhibiting scoliosis. Although we cannot exclude other possibilities, such as the involvement of hedgehog signaling during scoliosis, we think it is very likely that *kif7* mutants may display RF defects at later stages. Finally, it is worth mentioning that the role of cilia in regulating Hedgehog signaling is relatively minor in zebrafish as both *kif7* and cilia mutants displayed only minor defects in neural tube patterning (Huang & Schier, 2009, Lunt, Haynes et al., 2009, Maurya, Ben et al., 2013).

**Reference**

Cantaut-Belarif Y, Sternberg JR, Thouvenin O, Wyart C, Bardet PL (2018) The Reissner Fiber in the Cerebrospinal Fluid Controls Morphogenesis of the Body Axis. *Curr Biol* 28: 2479-2486 e4

Huang P, Schier AF (2009) Dampened Hedgehog signaling but normal Wnt signaling in zebrafish without cilia. *Development* 136: 3089-98

Lunt SC, Haynes T, Perkins BD (2009) Zebrafish ift57, ift88, and ift172 intraflagellar transport mutants disrupt cilia but do not affect hedgehog signaling. *Dev Dyn* 238: 1744-59

Maurya AK, Ben J, Zhao Z, Lee RT, Niah W, Ng AS, Iyu A, Yu W, Elworthy S, van Eeden FJ, Ingham PW (2013) Positive and negative regulation of Gli activity by Kif7 in the zebrafish embryo. *PLoS Genet* 9: e1003955

Olstad EW, Ringers C, Hansen JN, Wens A, Brandt C, Wachten D, Yaksi E, Jurisch-Yaksi N (2019) Ciliary Beating Compartmentalizes Cerebrospinal Fluid Flow in the Brain and Regulates Ventricular Development. *Curr Biol* 29: 229-241 e6

Terhune EA, Cuevas MT, Monley AM, Wethey CI, Chen X, Cattell MV, Bayrak MN, Bland MR, Sutphin B, Trahan GD, Taylor MRG, Niswander LA, Jones KL, Baschal EE, Antunes L, Dobbs M, Gurnett C, Appel B, Gray R, Hadley Miller N (2021) Mutations in KIF7 implicated in idiopathic scoliosis in humans and axial curvatures in zebrafish. *Hum Mutat* 42: 392-407

Zhang X, Jia S, Chen Z, Chong YL, Xie H, Feng D, Wu X, Song DZ, Roy S, Zhao C (2018) Cilia-driven cerebrospinal fluid flow directs expression of urotensin neuropeptides to straighten the vertebrate body axis. *Nat Genet* 50: 1666-1673
